# Memory T Cells in Flavivirus Vaccination

**DOI:** 10.3390/vaccines6040073

**Published:** 2018-10-18

**Authors:** Guangyu Li, Cody Teleki, Tian Wang

**Affiliations:** 1 Department of Microbiology & Immunology, University of Texas Medical Branch, Galveston, TX 77555, USA; guli@UTMB.EDU (G.L.); ccteleki@UTMB.EDU (C.T.); 2 Department of Pathology, University of Texas Medical Branch, Galveston, TX 77555, USA; 3 Institute for Human Infections & Immunity, University of Texas Medical Branch, Galveston, TX 77555, USA; 4 Sealy Institute for Vaccine Sciences, University of Texas Medical Branch, Galveston, TX 77555, USA

**Keywords:** flavivirus, memory T cells, vaccine

## Abstract

Flaviviruses include many medically important viruses, such as Dengue virus (DENV), Japanese encephalitis (JEV), tick-borne encephalitis (TBEV), West Nile (WNV), yellow fever (YFV), and Zika viruses (ZIKV). Currently, there are licensed human vaccines for DENV, JEV, TBEV and YFV, but not for WNV or ZIKV. Memory T cells play a central role in adaptive immunity and are important for host protection during flavivirus infection. In this review, we discuss recent findings from animal models and clinical trials and provide new insights into the role of memory T cells in host protective immunity upon vaccination with the licensed flavivirus vaccines.

## 1. Introduction

Flaviviruses are a group of small, enveloped, positive-stranded RNA viruses. The 11 kb genome encodes, translates, and is processed into three structural proteins—capsid (C), envelope (E), membrane (M)—and seven non-structural (NS) proteins; NS1, NS2A, NS2B, NS3, NS4A, NS4B, and NS5. The E protein, a major virion surface protein, is involved in receptor binding and membrane fusion, and induces neutralizing antibodies in the infected hosts. *Flavivirus* genus includes more than 70 RNA viruses, many of which are mosquito or tick-borne human pathogens, such as dengue virus (DENV), Japanese encephalitis virus (JEV), tick-borne encephalitis virus (TBEV), West Nile virus (WNV), yellow fever virus (YFV), and Zika virus (ZIKV) [1,2]. Flavivirus infection can cause a wide variety of clinical manifestations and complications in humans ranging from undifferentiated fever, hemorrhagic fever, encephalitis and death. To date, there are no specific anti-viral therapeutics [3,4]. Vaccination has been one major preventive measure against flavivirus infection.

During viral infection, innate immunity provides immediate control of virus replication and dissemination. In comparison, adaptive immunity (T cells and antibody-producing B cells) takes longer to develop, targets more specifically to viral antigens and plays a critical role in viral clearance and host recovery. Vaccination educates both innate and adaptive immune systems in order to boost adaptive T and B cell memory responses and provide rapid protection against subsequent infection with related viruses. Over the last seven decades, various strategies have been utilized to develop flavivirus vaccines. Currently, effective vaccines have been licensed for human use to combat YFV, JEV, DENV and TBEV infection. While it has been documented that neutralizing antibodies generated by these vaccines provide host protection, the role of T cell-mediated immunity is not yet fully understood. In this review, we focus on recent advances in understanding memory T cell responses to the licensed flavivirus (DENV, JEV, TBEV and YFV) vaccines, and the evidence of how they provide immune protection in humans or animal models.

## 2. Memory T Cell Responses to Flavivirus Infection

### 2.1. Memory T Cells

During viral infection, naïve T cells become activated when they encounter cognate antigen presented by antigen-presenting cells (APCs) and differentiate into effector T cells. After a peak of the effector responses, the majority of activated T cells die during contraction phase and the remaining 5–10% survive to acquire memory functions. Memory T cells recognize epitopes on conserved internal viral proteins, and respond more effectively following a secondary viral infection. They are central to all adaptive immune responses [5]. Memory T cells include central memory T cells (T_CM_), effector memory T cells (T_EM_), tissue resident memory T cells (T_RM_), and virtual memory T cells (T_VM_) [6,7,8,9,10,11]. CD4^+^ and CD8^+^ memory T cells express CD45RO [12]. T_CM_ stay in lymphoid organs and bone marrow and have a high proliferative potential. T_EM_ are located in peripheral tissues, which enable them with immediate action on pathogen recognition. T_RM_ settle within specific organs including intestines, lung, liver, spleen, brain, lymph node, skin and other mucosal surfaces, which play an important role on the protection against tissue-specific infections and mucosal infections [13,14]. T_VM_ are antigen-inexperienced, but antigen-specific T cells, which can reside outside of the naïve T cell compartment and relate to physiological homeostatic mechanisms. Through homeostatic proliferation, memory T cells may persist life-long, even without antigen exposure [6,7,15].

### 2.2. T Cell Immunity in Flavivirus Infection

Viral antigens are displayed on the surfaces of infected cells in the context of the major histocompatibility complex (MHC) Class I molecules, and are recognized by cytotoxic CD8^+^ T cells, which mediate the direct killing of virus-infected cells and produce anti-viral cytokines [16]. Meanwhile, CD4^+^ T cells recognize viral peptides associated with MHC class II, which is expressed by APCs. CD4^+^ T cells carry out multiple functions, including activation of B-lymphocytes, cytotoxic T cells, as well as nonimmune cells, and also play a critical role in the suppression of immune reaction. Neutralizing antibodies have been primarily associated with epitopes on the E protein. Most T cell epitopes have been mapped in flavivirus NS proteins [17,18]. B cells and specific antibodies are critical in the control of disseminated flavivirus infection [19,20]. Both CD4^+^ and CD8^+^ effector and memory T cells have been shown to directly contribute to host protective immune responses, including viral clearance, and providing help for B cells and antibody maturation [21,22,23,24,25,26,27]. For example, CD4^+^ effector memory T cells expressing CD45RA (TEMRA) are implicated in protective immunity against DENV infection. An enhanced magnitude and functionality of CD4^+^ TEMRA cells correlate with the human leukocyte antigen (HLA) allelic variants were associated with relative resistance to severe DENV diseases [28]. CD4^+^ cytotoxic T lymphocytes are known to protect host from DENV infection [29]. In another study [30], single-cell differential gene expression analysis revealed high levels of CD4^+^ cytotoxic T lymphocytes and precursor cells were enriched in the T_EMRA_ subset. Lastly, the development of the polyfunctional tissue resident CD8^+^ memory T cells in the central nervous system (CNS) helps to control virus dissemination and decrease WNV lethality [31,32].

## 3. Role of T Cells in Host Immunity against Flavivirus Vaccines

### 3.1. DENV

#### 3.1.1. DENV Infection

DENV has become one of the most important global public health threats in recent decades with nearly half the world’s population living at the risk. There are four genetically and antigenically distinct DENV serotypes, including DENV1, DENV2, DENV3 and DENV4. DENV-induced clinical diseases range from asymptomatic infection, dengue fever to a life-threatening hemorrhagic disease (dengue hemorrhagic fever (DHF) and dengue shock syndrome (DSS)) [33].

#### 3.1.2. DENV Vaccine Development

Currently, the only vaccine approved for human use is Dengvaxia (Table 1, CYD-TDV) developed by Sanofi Pasteur. CYD-TDV has been licensed in several countries, including Mexico, Brazil, El Salvador, Paraguay, and the Philippines. It is a recombinant live attenuated chimeric trivalent vaccine combination of four monovalent chimeric attenuated viruses that comprise the pre-membrane (prM) and E sequence of each DENV serotype grafted onto the NS protein backbone of YFV 17D [34]. The vaccine is known to elicit high neutralizing titers in vaccinees, but the overall protective efficacy across all four DENV serotypes was only 60.3% [35]. While CYD-TDV is considered a first-generation vaccine, more efforts have been made towards the development of novel second-generation vaccines with improvements on both the protective efficacy and feasible dosing regimen usable for travelers or endemic market. Among them, several are in the advanced stages of clinical development [35]. One candidate, a live-attenuated tetravalent dengue vaccine (Takeda, TDV) consists of an attenuated DENV-2 strain (TDV-2), and three chimeric viruses containing the prM and E genes of DENV1, DENV3, and DENV4 expressed in the context of the TDV-2 genome (TDV-1, TDV-3, TDV-4, respectively). TDV (under the previous name DENVax) has been developed by Takeda Vaccines Inc. It has been extensively tested in preclinical studies and clinical trials. Another second-generation vaccine candidate is the live attenuated TV003/TV005 vaccine developed by US National Institutes of Health (NIH), which is in phase III development. It has been licensed to Merck, and companies in Brazil (Instituto Butantan), India (Panacea Biotec and Serum Institute of India), and Vietnam (Vabiotech). The attenuation of the vaccine was done by producing recombinant DEN viruses that carry nucleotide deletion in the 3′ untranslated region (UTR) or that were chimerized between DENV [36].

#### 3.1.3. Memory T Cell Responses to DENV Vaccines

An early phase II trial in Singapore [37] revealed that the CYD-TDV vaccine induced YF-17D-NS3-specific CD8^+^IFNγ^+^ T cell responses, and CYD-specific T helper 1/T cytotoxic 1 cellular response in all participants with a predominant IFN-γ secretion compared with TNF-α and low levels of IL-13 following ex vivo re-stimulation of peripheral blood mononuclear cells (PBMC) with each the CYD vaccine viruses. Although DENV NS3-specific CD4^+^ T cell response pre-existed in adult participants, vaccination induced similar levels of CD4^+^TNF-α and IFN-γ responses. Although T cell responses were directed mainly against CYD-4 after the first vaccination, they were more balanced against all four serotypes after the third immunization and the memory T cell response persisted after one year following CYD-TDV vaccination with 2- to 3-fold lower NS3-specific, and serotype-specific T cell activities [37]. The persistence of E-specific IFN-γ secreting memory T cells was also reported in vaccinees 9 years after exposure to the live-attenuated vaccine [38].

In one clinical trial, Chu et al. [39] showed immunization with two doses of TDV vaccine 3 months apart triggered CD8^+^ T cells specific for the NS1, NS3, and NS5 proteins of TDV-2. In particular, CD8^+^ T cells were multifunctional and cross-reactive to NS proteins of the other three DENV serotypes. CD8^+^ T cell responses reached the peak levels on day 90 after the first dose and persisted 6 months following the second immunization. Animal studies also showed cross protection of the multifunctional CD8^+^ T cell response after vaccination with live attenuated DENV2 PDK53, the core component of the tetravalent TDV vaccine, though the vaccinated mice developed enhancing, interfering maternal antibodies at the same time [40].

A randomized double-blind, placebo-controlled clinical trial was conducted in recipients of TV003 or placebo followed by challenge 6 months later with a DENV-2 strain, rDEN2Δ30. All 21 recipients of TV003 who were challenged with rDEN2Δ30 were protected from infection with rDEN2Δ30. None developed viremia or any illness after challenge. In contrast, all 20 placebo recipients challenged with rDEN2Δ30 developed viremia, and some developed rash or neutropenia [41]. Vaccination with TV003 not only elicits protective neutralizing antibodies, but also the antigen-specific CD8^+^ T cell response which were readily detectable and comparable to natural DENV infection. T cell responses following tetravalent vaccination were, dramatically, focused toward the highly conserved NS protein. Broad responses to structural and NS proteins were observed after monovalent vaccination [42]. Furthermore, CD4^+^ T cells elicited by a tetravalent live attenuated DENV vaccine (TV005) recognize epitopes identified in natural infection, and dominantly recognize the capsid, NS2A, and NS5 proteins. The vaccine-specific CD4^+^ T cell responses are similar in magnitude, frequency, and specificity to those observed in humans naturally exposed to DENV, suggesting that it would induce a T cell response protective against severe disease [43]. A more recent study [44] shows that the vaccine rDEN2Δ30 induced stronger CD8^+^ T responses, but less CD4^+^ T cell responses than those to natural DENV2 infection, suggesting the differences between CD4^+^ and CD8^+^ T cell responses induced by vaccines compared to natural viruses infection. Furthermore, Paquin-Proulx, et al. [45] demonstrated that vaccination with the live, attenuated tetravalent vaccine TV003 induces a CD4^+^ T-cell response against ZIKV, providing evidence for T cell cross-reactivity between DENV and ZIKV. Memory T cell responses elicited by prior vaccination with TV005 also recognize ZIKV-specific peptides. This cross-reactivity can be explained by the sequence similarity of the two viruses. DENV exposure prior to ZIKV infection also influences the timing and magnitude of the T cell response. ZIKV-reactive T cells in the acute phase of infection were detected earlier and in greater magnitude in DENV-immune patients [46].

### 3.2. JEV

#### 3.2.1. JEV Infection and Immunity

The majority of JEV infections are asymptomatic; less than 1% of infections induce encephalitis. JE is the major viral encephalitis in South Asia and the Western Pacific [47]. Compared to other flaviviruses, JEV is more likely to induce severe neurological disease upon acute infection. The virus can also persist in the CNS and peripheral blood for several months after its initial acute infection [48,49]. Functional CD4^+^ and CD8^+^ T cell responses have been linked to different clinical outcomes of JEV infection. For example, JEV NS protein specific-CD8^+^ T cell responses were mostly observed in healthy JEV-exposed donors; whereas CD4^+^ T cell responses were noted in recovered JE patients, which targeted structural proteins and the secreted NS1 protein. Importantly, a high quality, poly-functional CD4^+^ T cell response was associated with a complete recovery from JE [50].

#### 3.2.2. Licensed JEV Vaccines

Several different vaccines have been developed to control JE since the 1950s. Currently, inactivated, live attenuated and chimeric vaccines are licensed for human use, and are efficacious against more than one strain and genotype (Table 1, [47]). For more than half a century, the mouse brain derived inactivated (MBDI) vaccine was the only vaccine available for human use. However, the production of MBDI vaccine was discontinued in 2005 due to its poor immunogenicity and adverse events [47]. The Vero cell-derived inactivated vaccine has replaced the MBDI. The live attenuated SA14-14-2 JEV vaccine is the mostly commonly used JEV vaccine. It was derived from attenuating passages of a wild-type virus [51].The vaccine is completely attenuated for mice, tolerable for human use in both adults and children and it does not replicate in mosquitoes [47]. Vaccines usage has resulted in a decrease in JE incidence in many Asian countries. Nevertheless, JE remains a major problem in Asia. In addition, a low seroconversion of neutralization antibody was reported for the JEV vaccine SA 14-14-2 [51].

#### 3.2.3. T Cell Responses to Licensed JEV Vaccines

The longevity of protective antibody responses induced by SA14-14-2 live-attenuated vaccine as well as the chimeric JEV vaccine have been well demonstrated [52,53]. The elicitation of T cell mediated immune responses and their long-term duration are not well understood yet. It has been shown that sublingual immunization with the live attenuated SA14-14-2 in mice induces JEV-specific IFN-γ^+^ CD4^+^ and CD8^+^ T cell responses [54]. In one clinical study in South India [55], T cell responses were evaluated following vaccination of 15 adults with a single dose of JEV live-attenuated vaccine SA14-14-2. Eighty-seven percent of participants produced IFN-γ^+^ T cell responses against JEV proteins. The inactivated vaccine was found to be less effective in protecting mice against JEV infection compared to the live attenuated vaccine [56]. High levels of IFN-γ^+^ CD4^+^ T cells were noted in female BALB/c mice vaccinated with JEV vaccine SA-14-14-2. In contrast, the inactivated vaccine only induced a limited immune response and partial protection, which may be due to the decreased activity of dendritic cells and the expansion of CD4^+^CD25^+^Foxp3^+^ regulatory T cells observed in vaccinated mice.

### 3.3. TBEV

#### 3.3.1. TBEV Infection and Vaccine Development

TBEV causes mild to moderate febrile illness in humans that may progress to encephalitis, leading to severe long-term complications and sometimes death [57]. Currently, licensed TBEV vaccines include the Western European vaccines FSME-Immun and Ecepur, the Russian TBE-Moscow vaccine, EnceVir and the Chinese vaccine (Changchun Institute of Biological Products, China) (Table 1, [58]). In 2004, a second-generation inactivated vaccine produced in a primary hamster kidney (PHK) cell line (SenTaiBao, Changchun Institute of Biological Products Co., Ltd., Jilin, China) was approved in China for the prevention of TBE [59]. The Western European and Russian vaccines contain formalin-inactivated TBEV and aluminum hydroxide as an adjuvant and are both used in adults and children [58,60]. There is limited data published on the direct efficacy data for the second-generation, PHK-inactivated vaccine.

#### 3.3.2. T Cell Immunity and Host Protection in TBEV Vaccinees

The mechanism of vaccine-induced protection against TBEV remains unclear. The formalin-inactivated tissue culture–derived virus vaccine is highly immunogenic and has been reported to induce protective neutralizing antibody responses after three doses [34]. Although neutralization antibodies stay persistently high 10 years following first booster vaccination [61], some animal studies showed that passive protection by neutralizing antibodies provide limited virus replication and protection from disease rather than sterilizing immunity [62]. While Eomes, Ki67 and T-bet identifies catalytic virus-specific CD8^+^ T cells in the peak effector stage of acute TBEV infection in patients; virus-specific CD8^+^ T cells transitioned to an Eomes^−^Ki67^−^T-bet^+^ population as the infection resolved and memory was established [63]. One week after intramuscular vaccination with the FSME-IMMUN, CD4^+^ and CD8^+^ central and effector memory T cells increased. However, the overall changes in the CD4^+^ and CD8^+^ T-cell subpopulations were small and did not show to have an impact on the distribution of the naïve and memory B-cell populations [64]. Further characterization of TBEV-specific CD4^+^ T cell response after an inactivated formalin-inactivated TBEV vaccine (FSME-Immun^®^ 0.5 mL, Baxter, Deerfield, IL, USA) in comparison to the response raised by natural infection were performed. Patients with TBEV infection had robust Th1 responses, producing IL-2, TNF-α and IFN-γ, which indicates that the responses are crucial for combating acute virus infection. In comparison, lower IFN-γ responses and high proportions of TNF-α^+^IL-2^+^T cells were noted following immunization with a formalin-inactivated TBEV vaccine [65]. Another study showed the magnitude of the TBEV-specific CD4^+^ T cell responses to capsid protein and E protein were significantly lower in patients with natural infection than in individuals immunized with an aluminum hydroxide-adjuvanted formalin-inactivated TBEV vaccine. This may be due to viral antagonism of the host immune response during infection or an enhanced T cell response after booster vaccination [66]. Overall, these studies suggest TBEV infection and vaccination induce differential T cell responses.

### 3.4. YFV

#### 3.4.1. YFV Infection and Vaccine Development

YFV is endemic to tropical and subtropical regions of South America and Africa. Although a majority of human YFV infections are asymptomatic, severe YF occurs in about 12% of infected individuals and may manifest with jaundice, hemorrhage, and multisystem organ failure [67]. The YFV 17D live-attenuated vaccine was developed by Max Theiler and colleagues in 1930s, for which he won the Nobel Prize in 1951. Three 17D sub-strains (17D-204, 17DD, and 17D-213) are used as vaccines (Table 1), which have minor differences in genome sequences, but all have proved to be effective vaccines [68].

#### 3.4.2. Immune Mechanisms of Host Protection by YFV 17D Vaccination

While it has been understood for many years now that YFV-17D vaccine elicits a strong humoral immune response, with neutralizing antibodies detectable in serum for over 30 years after vaccination, memory CD8^+^ T cells specific for the YFV-tetrameric antigen can also expand into effector pools at least 10 years after vaccination [69,70]. The mechanisms underlying YFV 17D efficacy have only been brought to light in the last several years. Recent evidence from animal models suggests that both humoral and cell-mediated immunity work in tandem to produce the lasting immunity seen following YFV 17D vaccination. Adoptive transfer of YFV-specific CD4^+^ T cells into naïve mice lacking the receptor to type I interferon before challenge with the Ang71 strain of YFV provided greater survival outcomes (5/8 survival rate) and less weight loss than mice that had received only CD8^+^ T cells (0/8 survival rate). However, when given serum from immunized mice in combination with adoptive transfer of CD4^+^ and CD8^+^ T cells, the mice challenged with Ang71 had 100% survival rates [71]. Bassi et al. [21] found that while YFV-specific memory CD8^+^ T cells were not required for protection against a challenge with YFV, they were trafficked into the brain with significantly higher kinetics than naïve CD8^+^ T cells. When testing for differences in CD4^+^ trafficking to the brain, memory CD4^+^ T cells were not found significantly earlier in the brain than naïve CD4^+^ T cells. Ad-vector encoding the NS3 protein from YF-17D could elicit a strong CD8^+^ T-cell response, which afforded a high degree of protection from subsequent intracranial challenge of the vaccinated mice [72]. Taken together, these studies point to a pivotal role of interactions between humoral and cell-mediated immunity in protecting against YFV infection.

#### 3.4.3. Memory T Cell Development Following YFV 17D Vaccination

In humans, CD4^+^ T cells seem to be biased towards a Th1-like response, as demonstrated by their rapid expression of CXCR3 following YFV 17D vaccination, and IFN-γ expression when re-exposed to YFV antigen [73]. These YFV-specific Th1-like cells remain detectable at least five years after vaccination. Utilizing predictive algorithm software, Zhang et al. [74] identified three nonameric epitopes from YFV NS4A and NS4B proteins capable of eliciting a robust IFN-γ^+^ CD3^+^CD8^+^ T cell response in YFV 17D-immunized mice. These epitopes were also found to be highly conserved across several strains of YFV, in addition to the YFV 17D vaccine strain. The effector functions of human CD8^+^ T cells during the course of YFV 17D infection have been characterized. Blom et al. [75] found a decline in polyfunctional effector CD8^+^ T cells between days 10, 14, and 90 post-infection, corresponding to peak CD4^+^, effector CD8^+^, and effector memory CD8^+^ T cell response respectively. Additionally, monofunctional CD8^+^ T cells expressed CD107a during peak CD4^+^ T cell response, but later switched to produce TNF-α as their effector molecule. While YFV-specific memory CD8^+^ T cells express similar surface molecules as naïve CD8^+^ T cells, such as CD45RA, CCR7, CD127, and CD28 (all of which are distinct from effector CD8^+^ T cells), memory T cells have significantly faster proliferative kinetics than the naïve cells [76].

Akondy et al. [77] found that the degree of effector CD8^+^ T cell expansion was related to the magnitude of viral load in 80 human volunteers. Those who experienced high viral load tended to have a larger effector CD8^+^ T cell response, and this was positively correlated with their memory CD8^+^ T cell response 90 days post-vaccination. Furthermore, in a murine model, Neves et al. [78] showed that higher initial IFN-γ production by γδ T cells in lymph nodes following vaccination with YFV 17D correlated with increased cytokine response by IFN-γ^+^CD4^+^ and IFN-γ^+^CD8^+^ cells. They also hypothesized that the larger initial IFN-γ response was due to increased viral replication within dendritic cells. Nevertheless, the clinical study conducted in African populations showed that an activated immune microenvironment including activation of CD8^+^ T cells and B cells as well as proinflammatory monocytes prior to vaccination are associated with impaired YFV-17D-induced CD8^+^ T cell responses [79]. Furthermore, a recent study showed the magnitudes of secondary responses induced by YFV-17D were much reduced compared to primary responses. In particular, the frequency and functional responses of total activated YFV-specific CD4^+^ and CD8^+^ T cells were reduced [80]. These results may argue that immunizing naïve individuals at risk is a priority and a more efficient use of available vaccine supplies. Collectively, these findings suggest both immune status prior to vaccination and virus replication contribute to memory T cell development upon vaccination. Further characterization of CD8^+^ memory T cells also revealed that the memory pool divided extensively during the first two weeks after infection and is maintained by quiescent cells that divide less than once every year. Unlike effector CD8^+^ T cells, memory CD8^+^ T cells do not produce the cytotoxic effector proteins granzyme B or perforin. However, patterns of CpG methylation at the granzyme B and perforin promoters did not significantly differ between the two cell populations, suggesting an epigenetic role in maintaining a lasting memory CD8^+^ T cells [76].

## 4. Conclusions

Although neutralizing antibody titer is the FDA-accepted primary endpoint of vaccine immunogenicity for flavivirus vaccines, increasing evidence suggests that neutralizing antibody only mildly correlates with protection. T cell mediated immunity may play a protective role in the absence of neutralizing antibody [76,81,82]. Clinical trials and animal studies of the currently licensed human flavivirus vaccines further support that T cells, in particular, multifunctional CD4^+^ and CD8^+^ memory T cell responses which have similar magnitude, frequency and specificity as those of natural infection contribute to a safe, efficacious and durable vaccine. In addition, immune status prior vaccination and epigenetic factors both contribute to the development and maintaining of memory T cells. These findings will likely provide new strategies for current development and licensure of WNV and ZIKV vaccines.

## Figures and Tables

**Table 1:** Licensed human flavivirus vaccines.

Virus	Vaccine Name	Vaccine Type	Developer
DENV	CYD-TDV (Dengvaxia)	Live attenuated	Sanofi Pasteur
	TDV (DENVax) *	Live attenuated	Takeda Vaccines Inc.
	TV003/005 *	Live attenuated	NIH
JEV	SA14-14-2	Live attenuated	BBIL, CDIBP
	JE-CV; ChimeriVax-JE	Chimeric	Acambis/Saofi Pasteur
	BK-VJE; JE-MB; JE-VC	Inactivated	BIKEN
	IXIARO	Inactivated	Valneva Austria GmbH
TBEV	FSME-Immun	Inactivated	Baxter
	Encepur	Inactivated	Novartis Vaccines
	TBE-Moscow	Inactivated	Federal State Enterprise of Chumakov Institute of Poliomyelitis and Viral Encephalitides; Russian Academy of Medical Sciences
	Encevir	Inactivated	Scientific Production Association Microgen
	TBE-PHK	Inactivated	Sen TaiBao, China
YFV	17DD	Live attenuated	Bio-Manguinhos (Fiocruz)
	17D-204	Live attenuated	Sanofi Pasteur; Institute Pasteur; Wuhan Institute of Biological Products, China
	17D-213	Live attenuated	Federal State Unitary Enterprise of Chumakov Institute, Russia

* Not licensed but at the advanced stages of clinical development.
